# Effects of enzymatic browning reaction on the usability of tobacco leaves and identification of components of reaction products

**DOI:** 10.1038/s41598-019-54360-2

**Published:** 2019-11-28

**Authors:** Yanjie Chen, Junfei Zhou, Ke Ren, Congming Zou, Junjun Liu, Guangmin Yao, Jianshen He, Gaokun Zhao, Wei Huang, Binbin Hu, Yi Chen, Kaisheng Xiong, Yan Jin

**Affiliations:** 1 Yunnan Academy of Tobacco Agricultural Sciences, Kunming, 650021 China; 2 College of Tobacco Science, Yunnan Agricultural University, Kunming, 650201 China; 3 School of Pharmacy, Tongji Medical College, Huazhong University of Science and Technology, Hubei, 430030 China

**Keywords:** Enzyme mechanisms, Plant molecular biology

## Abstract

The enzyme browning reaction results in grey speckles on tobacco leaves, which impairs the value and industrial usability of tobacco leaves. To demonstrate the influences of different browning degrees (BDs) of tobacco leaves on the usability of different cultivars and positions and identified structure of brown (grey) matter, we selected three flue-cured tobacco cultivars (K326, Yunyan87, and Honghuadajinyuan (Hongda)) and set four different BDs (<25%, 25% to 50%, 50% to 75%, and >75%). Indices related to: economic traits, chemical components, physical properties, and sensory quality of tobacco leaves with different cultivars were evaluated. Moreover, by utilising thin-layer chromatography and high-performance liquid chromatography, we analysed and identified the structure of the grey matter in terms of chemical composition. The experimental results show that the main component of grey speckles on tobacco leaves is 3-acetyl-6,7-dimethoxycoumarin (YC-ZJF). With the increase of BD, the amount of total sugar and reducing sugar, output value, the proportion of superior tobacco, shatter resistance index, and sensory evaluation score of the three cultivars significantly decrease, while the starch content increases significantly. The changes in protein, total nitrogen, and nicotine are insignificant with changing BD. In addition, other indices show different trends for different cultivars of flue-cured tobacco. After separation and identification of the components of grey speckled leaves, it is proved that the substance derived from grey speckles on tobacco leaves is YC-ZJF. The research is important to the study of browning mechanisms in tobacco leaves and provides corresponding targets for strategies to reduce browning thereof.

## Introduction

**Figure 1:**
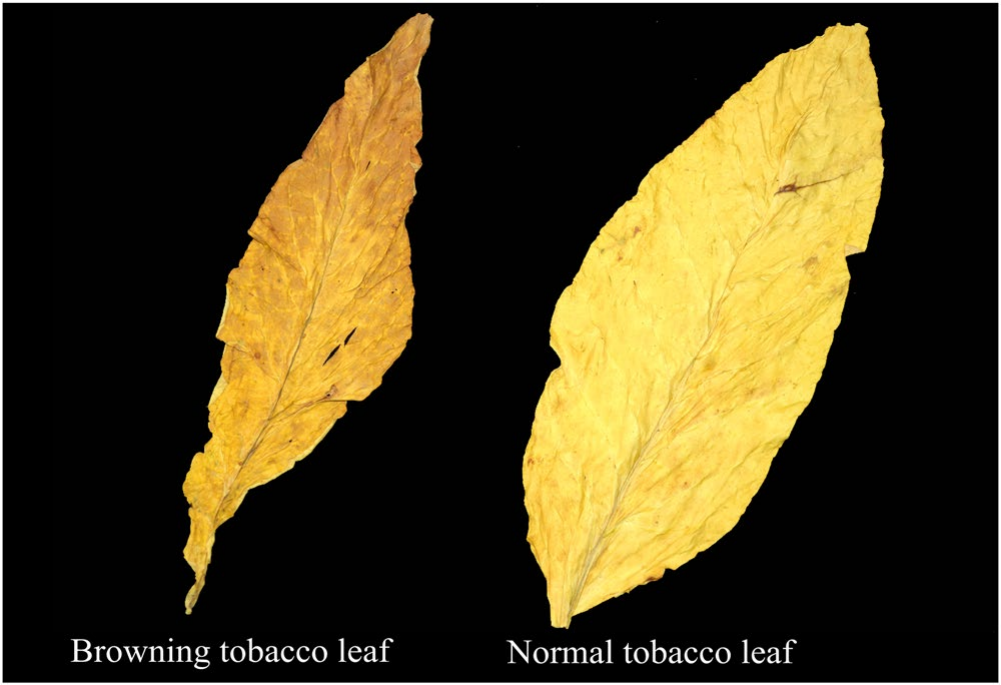
A comparison of the appearance of browning and normal flue-cured tobacco leaves.

Tobacco with grey speckled or browning leaves, in undesirable in flue-cured tobacco (Fig. 1) and it not only affects the supply of tobacco leaves, but also inhibits sustainable development of tobacco production; however, the influences of grey speckles on usability of tobacco leaves in different cultivars and positions and the structure of grey matters from enzymatic browning reactions remains unclear. Based on this, we aim to study the influences of different BDs of tobacco leaves (referring to the degree of deterioration of tobacco leaves with grey speckles) on the usability of tobacco leaves in different cultivars and positions and identified components of grey matter, to explore the browning mechanisms of grey speckled tobacco leaves.

Flue-cured tobacco has an artificial flue-curing process that takes 5–7 days^1^. Many changes occur in chemical composition and appearance of such leaves during flue-curing^2^. According to a survey of tobacco production, the rate of occurrence of tobacco leaves with grey speckle during flue-curing would be about 20 to 30%^3^. Tobacco leaves with grey speckle are inferior tobacco in accordance with tobacco leaf grading standards and it either is cheap or not purchased. It reduces tobacco quality and even deprives industrial usability of tobacco, which incurs economic loss among tobacco growers. If the rate of occurrence of tobacco leaves with grey speckle can be reduced by one percentage point by using scientific means, $42 million (USD) of average annual benefits can be obtained in tobacco-growing areas in Yunnan Province, China alone and 0.4 ha of cultivated land resource can be saved.

In the 1940s, Roberts^4^ proposed that an enzymatic browning reaction is responsible for appearance of grey speckles during flue-curing. During flue-curing, as the temperature rises to about 46 °C (temperature at which cell death occurs in most plants), the cytoplasmic membrane loses its selective permeability^5^. If cells are incompletely dehydrated at that temperature, polyphenol substances and polyphenol oxidase in cells can synthesise quinones, thus forming brown substances which manifest as grey tobacco^6,7^. Avoiding the formation of tobacco leaves with grey speckle has long been the research focus of scientific flue-curing and management. Drs Collins and Hawks^8^ proposed that attention should be paid to the coordination between pigment degradation and water abstraction at 42 to 48 °C in the early stage of drying tobacco leaves, so as to avoid tobacco leaves with grey speckle. If the water content in the leaves is very high, the temperature should be increased after losing 40% to 50% of the water in the leaves, instead of rashly increasing the temperature and delaying the yellowing stage to form grey speckles.

The previous research demonstrates that, in comparison with normal tobacco leaves, the amounts of all sugars and reducing sugar in tobacco leaves with moderate BD greatly decrease, while contents of nicotine, protein, and total nitrogen increase^9^. This leads to incongruous chemical components and a low sugar-alkali ratio. The amount of polyphenol substances in tobacco leaves is positively correlated with the quality, aroma, and taste of tobacco and chlorogenic acid and rutin are polyphenol substances exerting significant influences therein^10^. During flue-curing, the enzymatic browning reaction is one of reasons behind the reducing content of polyphenol substances in tobacco leaves^2,11,12^. Once tobacco leaves become completely brown, polyphenol substance contents can decrease by 85%, so that the aroma and taste of tobacco leaves deteriorate and the amount, and concentration, of offensive-odour components in tobacco leaves increase^13^. Grey speckles on tobacco leaves might not only affect appearance and quality to reduce the commodity value of tobacco leaves, but also result in improper and incongruent concentrations of chemical components in tobacco leaves, thus reducing aroma, taste, and industrial usability.

Tobacco leaves with grey speckles can appear in flue-curing process, and the poorer quality of some fresh tobacco can result in tobacco with grey speckled leaves. For example, when the contents of Fe^2+^ and Mn^2+^ in soils are high, the excess accumulation of Fe^2+^ and Mn^2+^ ions in tobacco can cause physical intoxication, thus generating tobacco leaves with grey speckles^14^. If only nitrogen fertiliser is applied, a nutritional imbalance in tobacco plants can induce immature tobacco to reach the required recovery standard. Tobacco injured by chilling due to a sudden decrease of temperature in its maturation period, overripe tobacco, tobacco with disease and tobacco with mechanical injury can also readily develop grey speckles during flue-curing^15,16^. Although mechanisms of formation of tobacco with grey speckles have been proposed for many years, there is no report of the chemical components and structure of grey matter generated in this browning reaction. The research mainly focusses on regulating the content of polyphenol substances and inhibiting the activity of polyphenol oxidase;^17,18^ however, some core mechanisms underpinning the enzymatic browning reaction in flue-curing remain unclear.

The influences of BD on the usability of tobacco leaves in different cultivars and positions need to be explored and components and structure of grey matters must be clarified. For these objectives, this experiment compared the influences of different BDs on physical and chemical properties, economic value, industrial usability of different cultivars of tobacco, and identified the structure of components of grey matter in browning tobacco. On this basis, this study attempts to enrich enzymatic browning mechanisms for tobacco during flue-curing, providing theoretical bases for reducing the incidence of tobacco with grey speckles during flue-curing, implementing strategies to help tobacco growers to increase income and promote sustainable development of the tobacco industry.

## Materials and Methods

### Experimental location and materials

The experiment was conducted in Shilin County (N24°46′27.55″, E103°17′18.83″), Kunming City, Yunnan Province, China with a prevailing plateau and mountain monsoon climate. The mean annual temperature is 16 °C and average annual rainfall is 939.5 mm. The average annual sunlight is 2,096.8 h. The three main local cultivars (K326, Yunyan87, and Hongda cultivars) were used in the experiment.

### Field management

Hongda, K326, and Yunyan87 cultivars were produced by utilising high-quality, high-efficiency cultivation techniques to cultivate tobacco with balanced nutrition, normal growth, and fresh leaves yellowed and matured layer-by-layer. In August, after cultivating for 90 to 95 d and topping for 35 to 40 d, tobacco leaves became pale yellow and 80% of leaves were yellowed, showing white, bright main veins, white branch veins, and downward rolled leaf apices and leaf margins. When leaves were wrinkled, mature leaves were collected on five to seven occasions at different positions and flue-cured according to the requirements for experimental design. Other agronomic practices were carried out following guidelines recommended by the Integrated Technology Promotion Centre at the Yunnan Academy of Tobacco Agricultural Sciences^19^.

### Experimental design

Fresh tobacco leaves with the same quality were selected for flue-curing in the furnace and then different BDs were set by suddenly reducing the temperature in the flue-curing process. Four different treatments, namely, BDs lower than 25%, in the ranges of 25% to 50%, 50% to 75%, and higher than 75% were set. After flue-curing K326, Yunyan87, and Hongda cultivars, tobacco leaves with the four BDs were used as samples for the experiment. By using local graders for tobacco leaves to grade the samples according to the National Standard for Flue-cured Tobaccos (GB 2635–1992), the appearance quality and economic traits of the three cultivars of treated tobacco samples were obtained. Some tobacco samples were sent to the laboratory of the Yunnan Academy of Tobacco Agricultural Sciences to determine physical indices and analyse chemical components, while the other samples were sent to the Technology Centre, China Tobacco Yunnan Industrial Co., Ltd for evaluation and rating. Meanwhile, after collecting grey matter from tobacco samples subjected to different treatments, the samples were sent to the College of Pharmacy, Huazhong University of Science and Technology (Key Laboratory of Natural Medicinal Chemistry and Resource Evaluation in Hubei Province) to identify their structure and components.

### Analyses

#### Economic traits

The raters graded the tobacco samples for the experiment according to National Standard (GB2635–92). The proportion of superior tobacco was estimated based on the national purchasing data and supervision and inspection data of grade quality during industry-commerce handover in that year. The flue-cured tobacco plants have distinct leaf numbers and weights due to different production areas and cultivars. For convenience, the number of leaves was estimated by using method proposed by Yan *et al*.^20^, thus allowing calculation of yield and output value of tobacco leaves.

#### Chemical components

The starch content was determined by spectrophotometry at 660 nm with HClO_4_ extraction^21^. Total sugar and reducing sugar were determined by rapid colorimetric method with 3,5-dinitrosalicylic acid^22^. The content of protein was determined with continuous flow analytical method^10^. Total nitrogen determined was using an elemental analysis method^23^. Nicotine was determined using a spectrophotometric method^24^. The polyphenol content was determined with HPLC coupled with ESI-MS after solid-phase extraction^25^.

#### Industrial machinability of tobacco leaves

After removing green miscellaneous tobacco from flue-cured tobacco, 10 kg samples of C3F and C2F were selected and sent to the Technology Centre, China Tobacco Yunnan Industrial Co., Ltd for analysing the industrial machinability. After equilibration for 72 h at constant temperature (22 °C) and humidity (60%), the samples were analysed according to the method for detecting the shatter resistance index of tobacco leaves provided by Chen *et al*.^26^.

#### Sensory quality

The sensory quality of the tobacco samples was evaluated by seven certified experts from the Technology Centre, China Tobacco Yunnan Industrial Co., Ltd according to 11 indices: the original aroma, aroma volume, aroma quality, concentration, biting taste, physiological strength, offensive odour, cleanliness, wetness, and aftertaste. The evaluation results refer to Sensory Technical Requirements for Cigarettes (GB5606.4–2005) for evaluating the sensory quality of tobacco leaves and nine-point scale was used to score these 11 indices. The data shown in this study are the mean of seven reports.

### Identification of main chemical components of grey matter in tobacco with grey speckles

#### Sample preparation for analysis using thin-layer chromatography

By separating the parts with grey speckles from parts without grey speckles on tobacco leaves, 50 g of the tobacco samples with grey speckles and 50 g without grey speckles were obtained. After crushing, they were extracted using ethanol at a concentration of 95%.

#### Extraction and isolation of the main chemical components of the tobacco with grey matter

**Figure 2:**
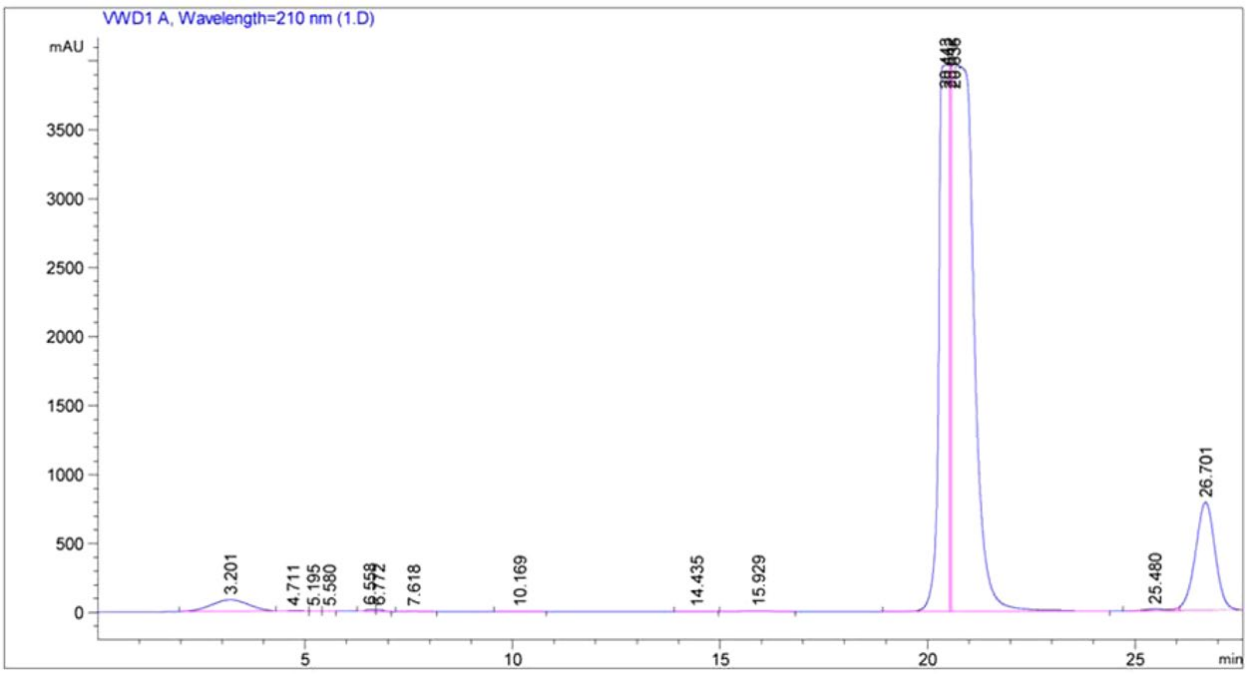
High-performance liquid chromatogram of component Fr.Ab1a.

After drying and crushing, the leaves of tobacco with grey matters were extracted with 95% ethanol four times (each for 3 d) at room temperature. The ethanol was removed under the reduced pressure to obtain 950 g residue. The extracts were separated and divided into three fractions (Fr.A, Fr.B, and Fr.C) by utilising silica gel (100 to 200 mesh) column chromatography (CC) eluted with a graduate methylene chloride/methanol solvent system. Fr.A (120 g) was further separated to three sub-fractions (Fr.Aa, Fr.Ab, and Fr.Ac) by silica gel CC eluted with methylene chloride/methanol (8:1). Fr.Ab (25 g) was separated using a reversed-phase C18 column eluted with a methanol/water system to yield three sub-fractions (Fr.Ab1, Fr.Ab2, and Fr.Ab3). Fr.Ab1 (4 g) was separated and purified by using Spephadex LH-20 (MeOH) to obtain a fraction Fr.Ab1a. Fr.Abla (220 mg) was finally purified by a high-performance liquid chromatograph [YMC-pack ODS-A column (5 μm, 10 × 250 mm), 35% MeCN/H_2_O in a flow rate of 2 mL/min] to yield 9.7 mg YC-ZJF (*t*_R_ = 20 min) (Fig. 2).

(YMC-pack ODS-A columns with a particle size of 5 μm and dimensions of 10 × 250 mm and 35% MeCN/H_2_O solution at flow rate of 2 mL/min were used.)

#### Statistical analysis

The data were analysed by using the general linear model (GLM) program of the SAS 9.3 computer package made by the SAS Institute Inc., Cary, NC. The statistical analysis of data in this study was based on the significance level of *P* < 0.05. Through Tukey’s (HSD) test, the mean of data was divided into 95% confidence intervals. Sigma Plot 12.3 (Systat Software Inc., Chicago, IL, USA) was used for plotting.

## Results

### Effects of different BDs on economic traits of different cultivars of flue-cured tobacco

**Figure 3:**
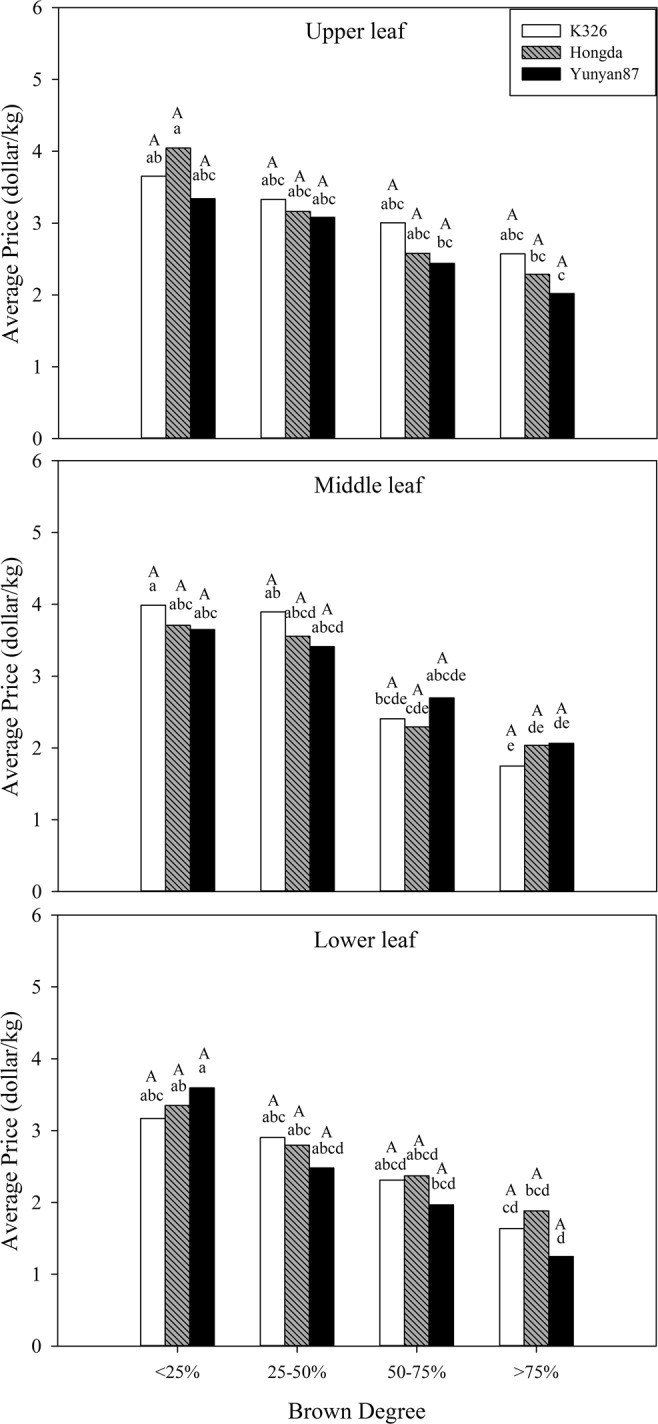
Effects of different BDs on the average price in flue-cured tobacco cultivars. Note: Different lowercase letters represent significant differences in proportions of superior tobacco between combinations of different cultivars and BDs in the same position. Different capital letters indicate significant differences in proportions of superior tobacco at different positions under combinations of the same cultivar and BD.

As shown in Table 1, BD and position exert significant effects (*P* < 0.05) on the average price and the proportion of superior tobacco. For the proportion of superior tobacco, BD has a synergistic effect with position. Figure 3 demonstrates that the average price with BD < 25% shows significant differences with BD > 75% in middle and upper leaves of the three flue-cured tobacco cultivars.

**Table 1:** Analysis of variance for the effects of BD, cultivar, and position with their interactions on economic traits.

Effect/contrast	DF	Average price	Proportion of superior tobacco	Output value
--------------Probability of a greater *F* value------------				
Browning degree(B)	3	<0.0001	<0.0001	<0.0001
Variety(V)	2	0.1037	0.3267	0.0001
Position (P)	2	<0.0001	<0.0001	<0.0001
BV	6	0.8928	0.8728	0.5202
BP	6	0.1425	0.0177	<0.0001
VP	4	0.4851	0.9529	0.0942
BVP	12	0.5637	0.9905	0.7949

**Figure 4:**
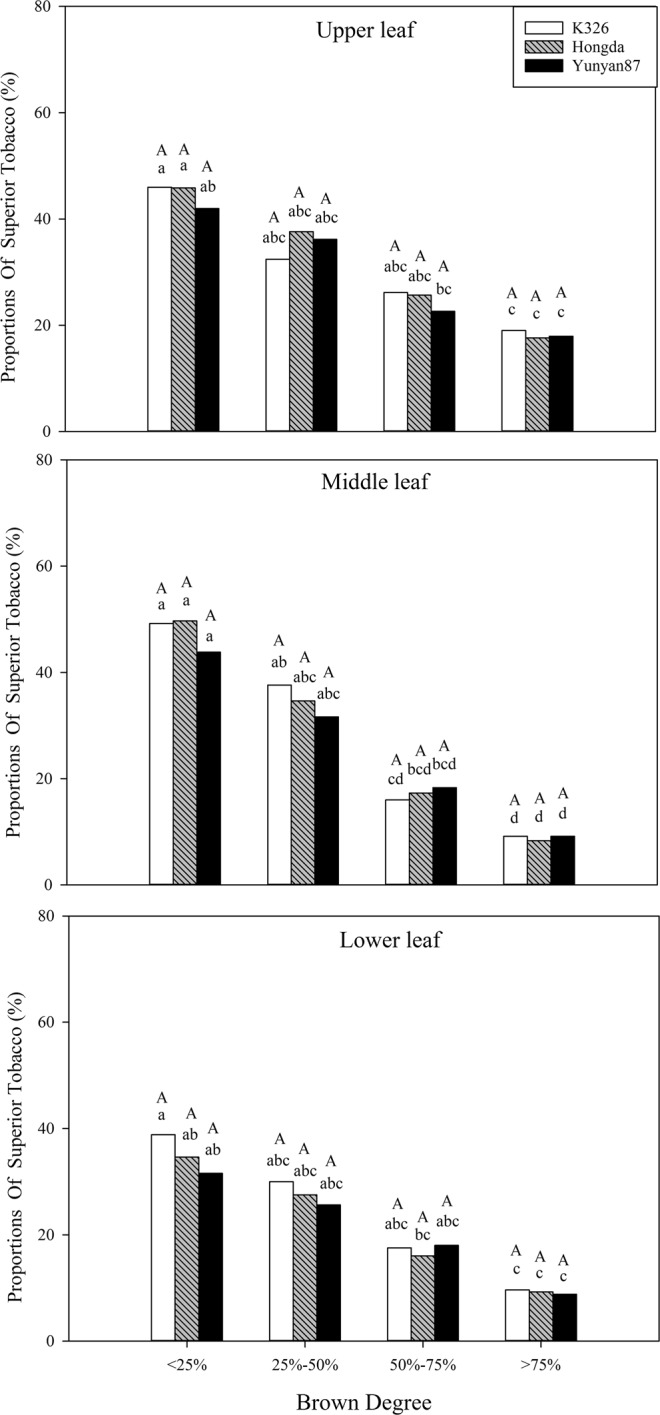
Effects of different BDs on the proportions of superior tobacco in flue-cured tobacco cultivars. Note: Different lowercase letters represent significant differences in proportions of superior tobacco between combinations of different cultivars and BDs in the same position. Different capital letters denote the significant differences in proportions of superior tobacco at different positions under combinations of the same cultivar and BD.

Figure 4 demonstrates that the proportions of superior tobacco in different positions of the three flue-cured tobacco cultivars reduce with increasing BD. At the same BD, the proportions of superior tobacco in different positions of each cultivar have no significant difference.

**Figure 5:**
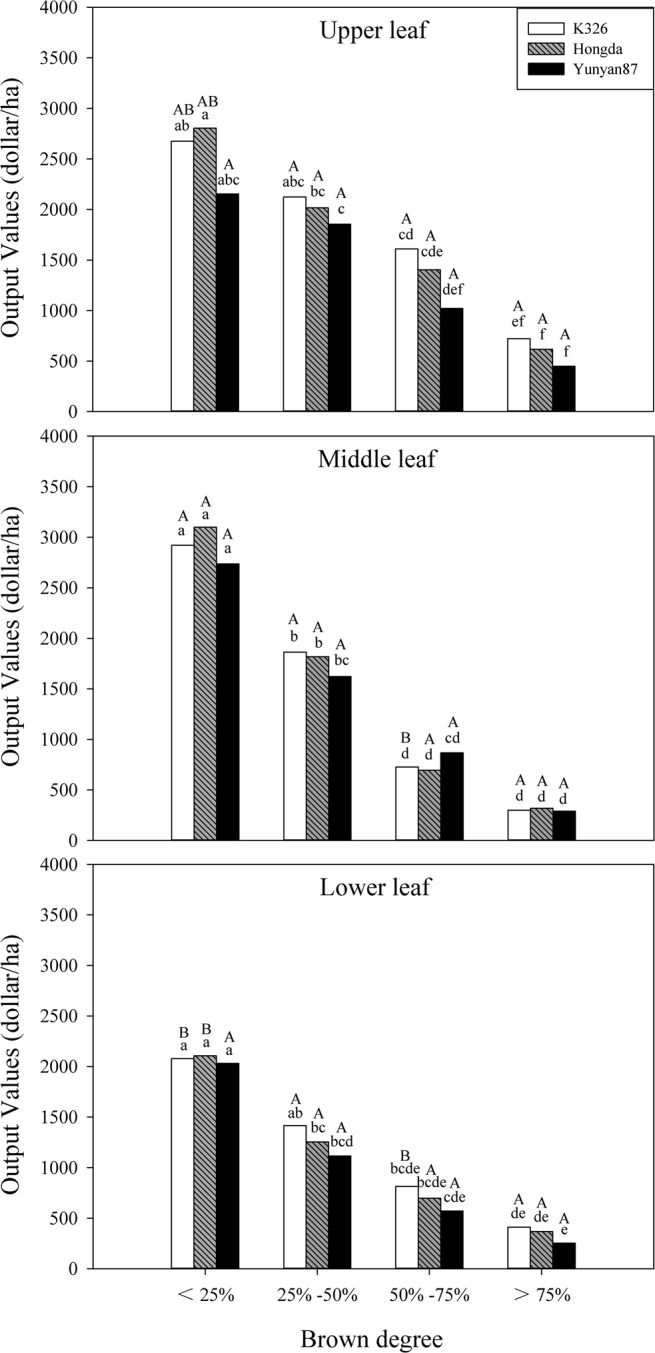
Effects of different BDs on output values of flue-cured tobacco cultivars. Note: Different lowercase letters represent significant differences in output values of tobacco leaves in the same position between combinations of different cultivars and BDs. Different capital letters indicate the significant differences in output values of tobacco leaves at different positions under combinations of the same cultivar and BD.

As shown in Table 1, BD, cultivar and position significantly affect output value (*P* < 0.05) and the interaction between BD and position has significant influences on output value (*P* < 0.05). Moreover, BD shows no synergistic effects with cultivar and position. Figure 5 shows that output values of tobacco leaves in different positions of each cultivar decrease with increasing BD. Under the interaction between cultivar and BD, output values of lower and middle leaves of K326 and Hongda cultivars with BD < 25% are significantly different. The output values of three flue-cured tobacco cultivars with BD < 25% show significant differences to those with BD > 75% in each position.

### Effects of different BDs on chemical characteristics of different cultivars

#### Effects of different BDs on starch content

**Figure 6:**
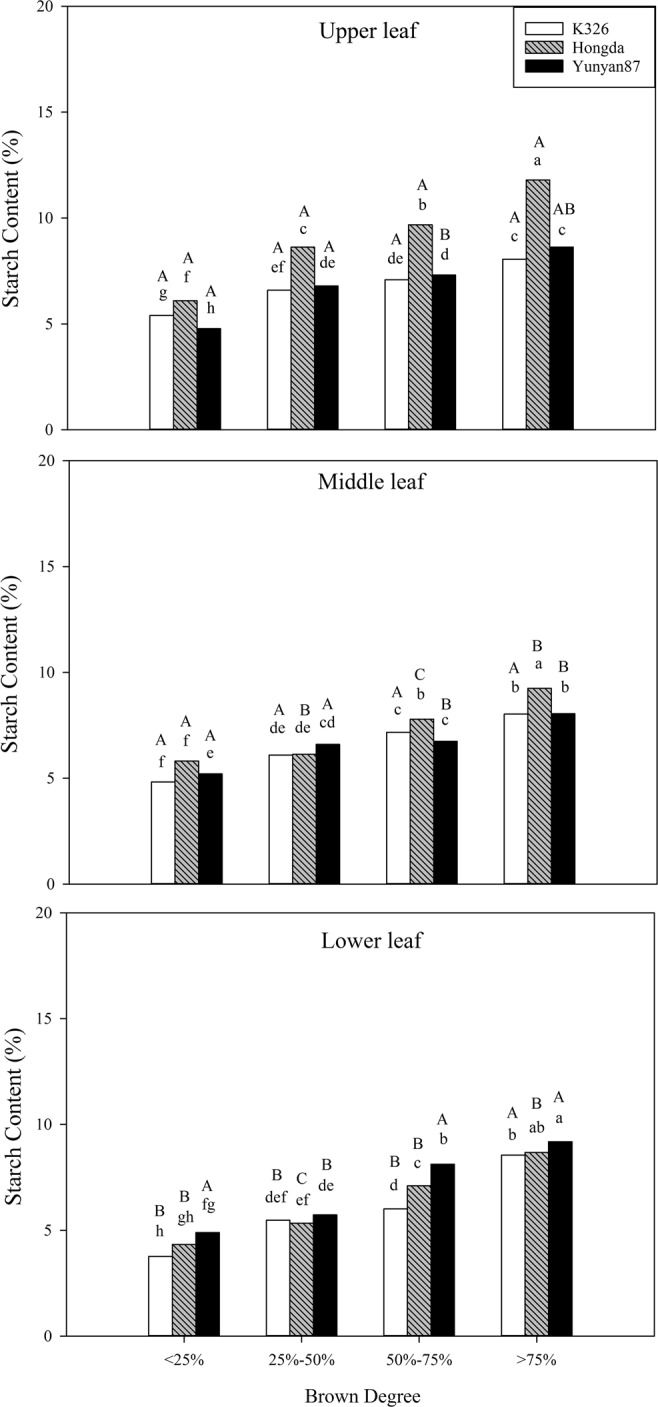
Effects of different BDs on starch contents in flue-cured tobacco cultivars. Note: Different lowercase letters represent significant differences in starch contents in tobacco leaves at the same position under combinations of different cultivars and BDs. Different capital letters indicate the significant differences in starch contents in tobacco leaves at different positions under combination of the same cultivar and BD.

According to Table 2, BD, cultivar, position, and their interaction demonstrate significant influences on the starch content (*P* < 0.05). Figure 6 shows that, with increasing BD, the starch contents all rise in tobacco leaves at each position of the three cultivars. At the same BD, when BD is smaller than 25%, the starch contents in lower leaves of K326 and Hongda cultivars are significantly lower than those in middle and upper leaves. When BD ranges from 25% to 50%, starch contents in lower leaves of K326 and Yunyan87 cultivars are significantly lower than those in middle and upper leaves; at each position, the Hongda cultivar shows significant differences in starch contents. When the BD changes from 50% to 75%, starch contents in lower leaves of K326 cultivar are significantly lower than those in middle and upper leaves. Starch contents at each position of the Hongda cultivar are significantly different, starch contents in lower leaves of the Yunyan87 cultivar are significantly higher than those in middle and upper leaves. When the BD exceeds 75%, starch contents in middle and lower leaves of Hongda cultivar are significantly lower than that in the upper leaves, while those in the middle and lower leaves of Yunyan87 cultivar are significant.

**Table 2:** Analysis of variance for the effects of BD, cultivar and position and their interactions on chemical components.

Effect/contrast	DF	Starch	Total sugar	Reducing sugar	Protein	Total nitrogen	Nicotine	Total polyphenols
------------------------------Probability of a greater *F* value------------------------------								
Browning degree (B)	3	<0.0001	<0.0001	<0.0001	<0.0001	<0.0001	0.2431	<0.0001
Variety (V)	2	<0.0001	<0.0001	<0.0001	<0.0001	<0.0001	<0.0001	<0.0001
Position (P)	2	<0.0001	<0.0001	<0.0001	<0.0001	<0.0001	<0.0001	<0.0001
BV	6	<0.0001	<0.0001	<0.0001	<0.0001	<0.0001	0.0437	0.0003
BP	6	<0.0001	<0.0001	0.0027	<0.0001	<0.0001	0.4859	<0.0001
VP	4	<0.0001	<0.0001	<0.0001	<0.0001	<0.0001	<0.0001	<0.0001
BVP	12	<0.0001	<0.0001	<0.0001	<0.0001	<0.0001	0.2211	0.0002

#### Effects of different BDs on the contents of total sugar

**Figure 7:**
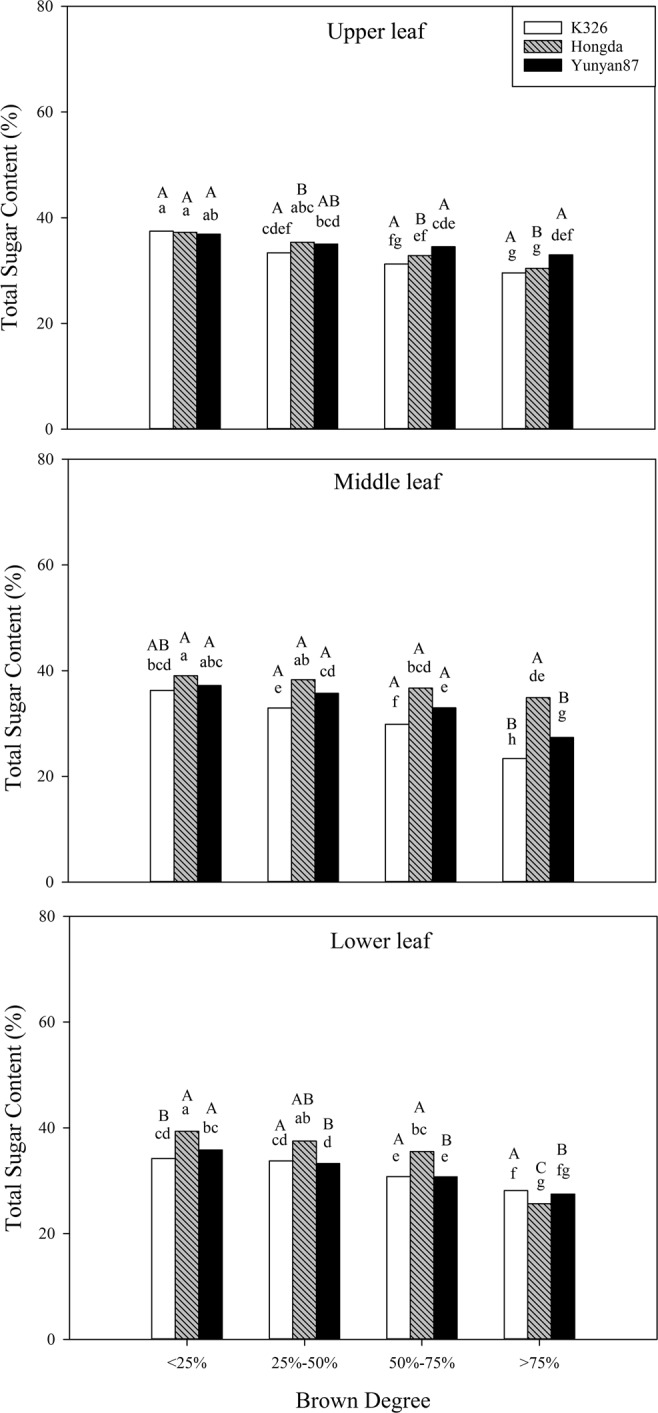
Effects of different BDs on the contents of total sugar in flue-cured tobacco cultivars. Note: Different lowercase letters represent significant differences in contents of total sugar in tobacco leaves at the same position between combinations of different cultivars and BDs. Different capital letters indicate the significant differences in contents of total sugar in tobacco leaves at different positions under combinations of the same cultivar and BD.

Based on Table 2, BD, cultivar, position and their interaction significantly affect the total sugar content (*P* < 0.05). Figure 7 shows that, as the BD increases, the total sugar contents in tobacco leaves at each position of different cultivars decrease. At the same BD, when the BD is smaller than 25%, lower and upper leaves of K326 exhibit significant differences in total sugars content: when the BD is between 25% and 75%, the total sugar content in lower leaves of K326 shows significant differences with those in middle and upper leaves. For the Hongda cultivar, the total sugar content in middle leaves is significantly higher than that in upper leaves. When the BD is higher than 75%, the total sugar contents in upper leaves of K326 and Yunyan87 cultivars are significantly higher than those in middle and lower leaves. Meanwhile, significant differences are found in contents of total sugar in leaves at different positions of Hongda cultivar.

#### Effects of different BDs on reducing sugar content

**Figure 8:**
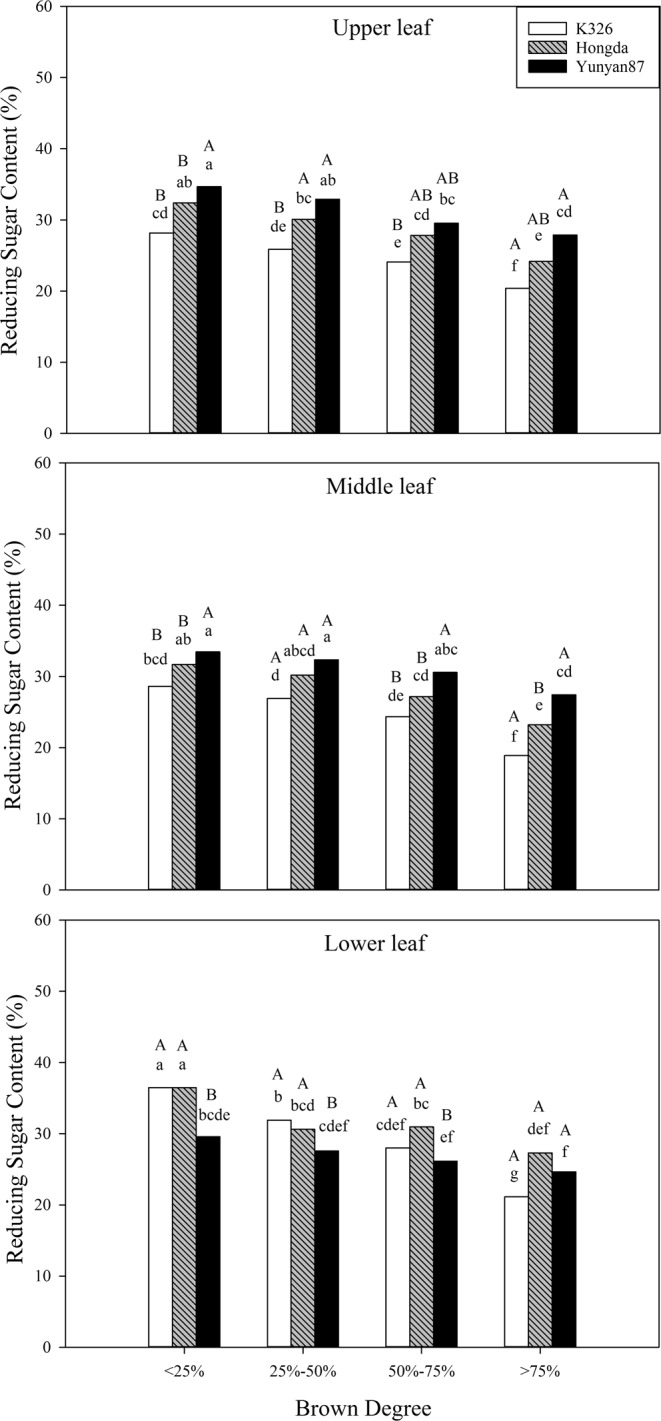
Effects of different BDs on the contents of reducing sugar in flue-cured tobacco cultivars. Note: Different lowercase letters represent significant differences in contents of reducing sugar in tobacco leaves at the same position under combinations of different cultivars and BDs. Different capital letters represent the significant differences in contents of reducing sugar in tobacco leaves at different positions under combinations of the same cultivar and BD.

Based on Table 2, BD, cultivar, position, and their interactions have significant influence on the reducing sugar content (*P* < 0.05). Figure 8 shows that the reducing sugar content in tobacco leaves in different positions of the three cultivars decreases with increasing BD. The reducing sugar contents in middle and upper leaves of Hongda and Yunyan87 cultivars are relatively high, while those in lower leaves of Hongda and K326 cultivars are high. When the BD is the same, namely BD < 25%, the lower leaves of the three cultivars show significant differences in reducing sugar content of their middle and upper leaves. When the BD ranges from 25% to 50%, the reducing sugar contents in upper leaves are significantly different from those in middle and lower leaves of the K326 cultivar, and the upper and middle leaves of Yunyan87 show significant differences therein to lower leaves. At a BD of 50% to 75%, the upper and middle leaves of the K326 cultivar demonstrate significant differences in the reducing sugar content with lower leaves, and middle and lower leaves of Hongda and Yunyan87 cultivars have significant differences therein. When the BD is higher than 75%, the reducing sugar contents in middle and lower leaves of the Hongda cultivar are significant.

#### Effects of different BDs on protein contents

**Figure 9:**
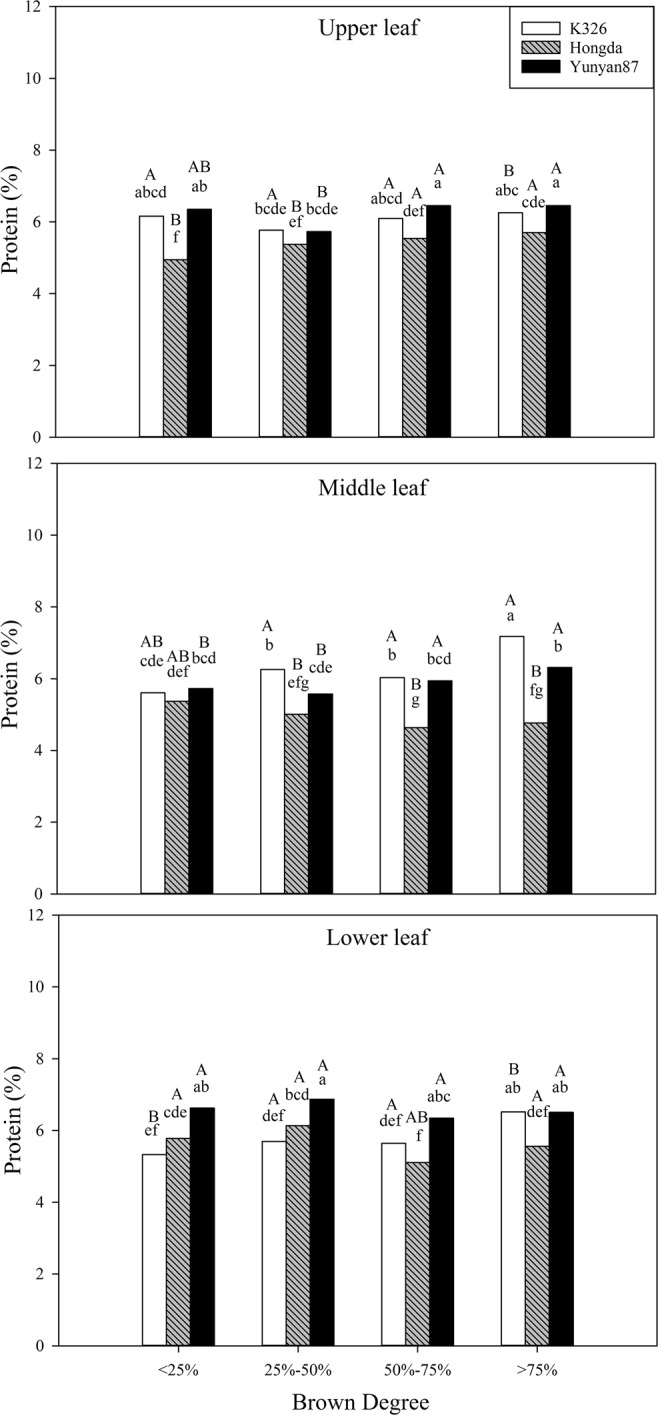
Effects of different BDs on protein contents in flue-cured tobacco cultivars. Note: Different lowercase letters represent significant differences in protein contents in tobacco leaves at the same positions under combinations different cultivars and BDs. Different capital letters indicate the significant differences in protein contents in tobacco leaves at different positions under combination of the same cultivar and BD.

In accordance with Table 2, BD, cultivar, position, and their interaction have significant effects on protein content (*P* < 0.05). Figure 9 shows that, when the BD is smaller than 25%, protein contents in upper and lower leaves of the Hongda and K326 cultivars are significantly different, and the protein contents of Yunyan87 cultivar in lower leaves show significant differences with that in the middle leaves. When the BD ranges from 25% to 50%, protein contents in lower leaves of Hongda and Yunyan87 cultivars are significantly higher than those in middle and upper leaves. When BD is between 50% and 75%, the differences between protein contents in upper and middle leaves of the Hongda cultivar are significant. When the BD exceeds 75%, protein contents in middle leaves of the K326 cultivar are significantly higher than those in upper and lower leaves; middle leaves of the Hongda cultivar contain significantly lower protein contents than upper and lower leaves.

#### Effects of different BDs on the total nitrogen content

**Figure 10:**
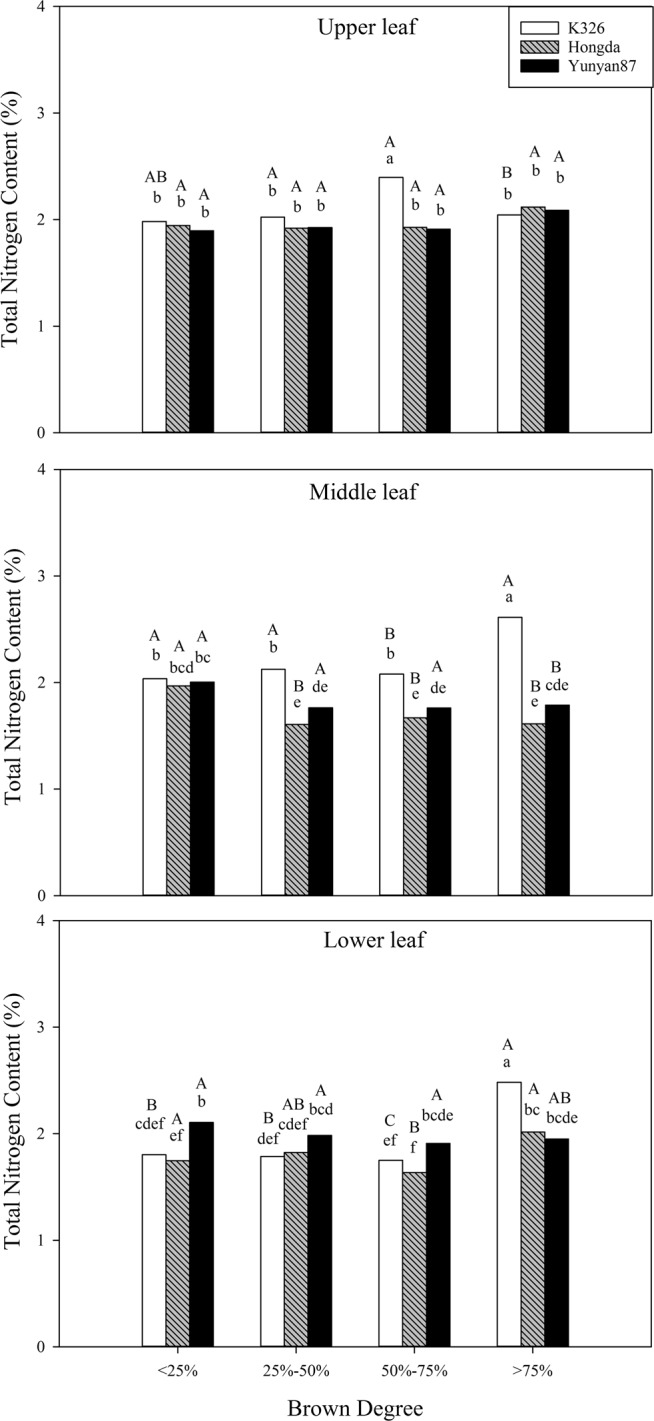
Influences of different BDs on total nitrogen contents in flue-cured tobacco cultivars. Note: Different lowercase letters represent significant differences in total nitrogen contents in leaves at the same position under combinations of different cultivars and BDs. Different capital letters indicate the significant differences in total nitrogen contents in leaves at different positions under combinations of the same cultivar and BD.

According to Table 2, BD, cultivar, position, and their interaction show significant effects on the total nitrogen content (*P* < 0.05). Figure 10 shows that, at the same BD of less than 25%, total nitrogen contents in lower leaves of the K326 cultivar are significantly lower than that in middle leaves. When BD varies from 25% to 50%, the total nitrogen contents in middle and upper leaves of the K326 cultivar are significantly higher than that in lower leaves. When the BD is in the range of 50% to 75%, significant differences in total nitrogen contents are found in each position of the K326 cultivar, while the total nitrogen contents in upper leaves of the Hongda cultivar are significantly higher than those in middle and lower leaves. When the BD is higher than 75%, the total nitrogen contents in upper leaves of the K326 cultivar are significantly lower than those in middle and lower leaves. The total nitrogen contents in middle leaves of the Hongda cultivar are significantly lower than those in upper and middle leaves.

#### Effects of different BDs on nicotine content

**Figure 11:**
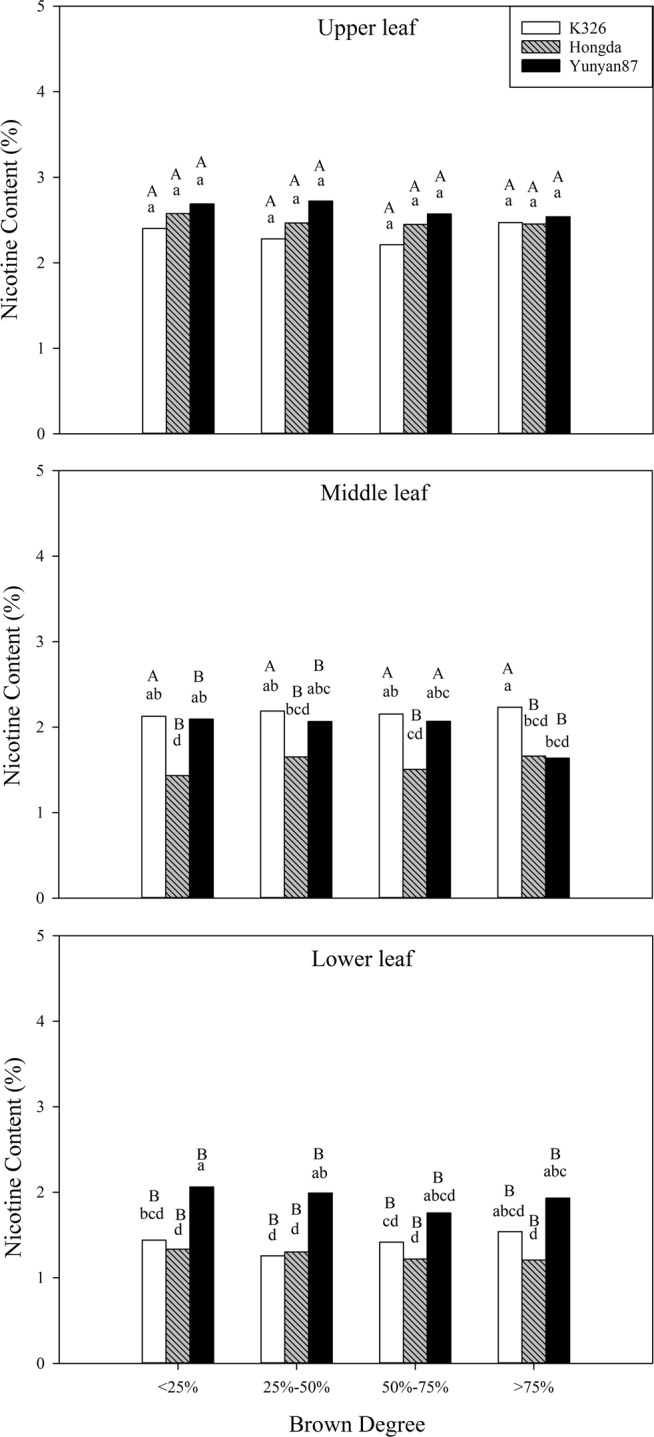
Effects of different BDs on nicotine contents in flue-cured tobacco cultivars. Note: Different lowercase letters represent significant differences in nicotine contents in tobacco leaves at same positions under combinations of different cultivars and BDs. Different capital letters indicate the significant differences in nicotine contents in tobacco leaves at different positions under combinations of the same cultivar and BD.

In accordance with Table 2, cultivar and position, as well as their interaction exert significant influences on nicotine content (*P* < 0.05) and interaction between BD and cultivar also significantly affects the nicotine content. Figure 11 shows that, at the same BD of less than 25%, the nicotine contents in lower leaves of the K326 cultivar are significantly lower than those in middle and upper leaves, while nicotine contents in middle and lower leaves of the Hongda and Yunyan87 cultivars are significantly lower than that in upper leaves. When BD ranges from 50% to 75%, nicotine contents in lower leaves of the K326 and Yunyan87 cultivars are significantly lower than those in middle and upper leaves. At other BDs, nicotine contents in middle and lower leaves of the Yunyan87 cultivar are significantly lower than that in upper leaves.

#### Effects of different BDs on polyphenol content

**Figure 12:**
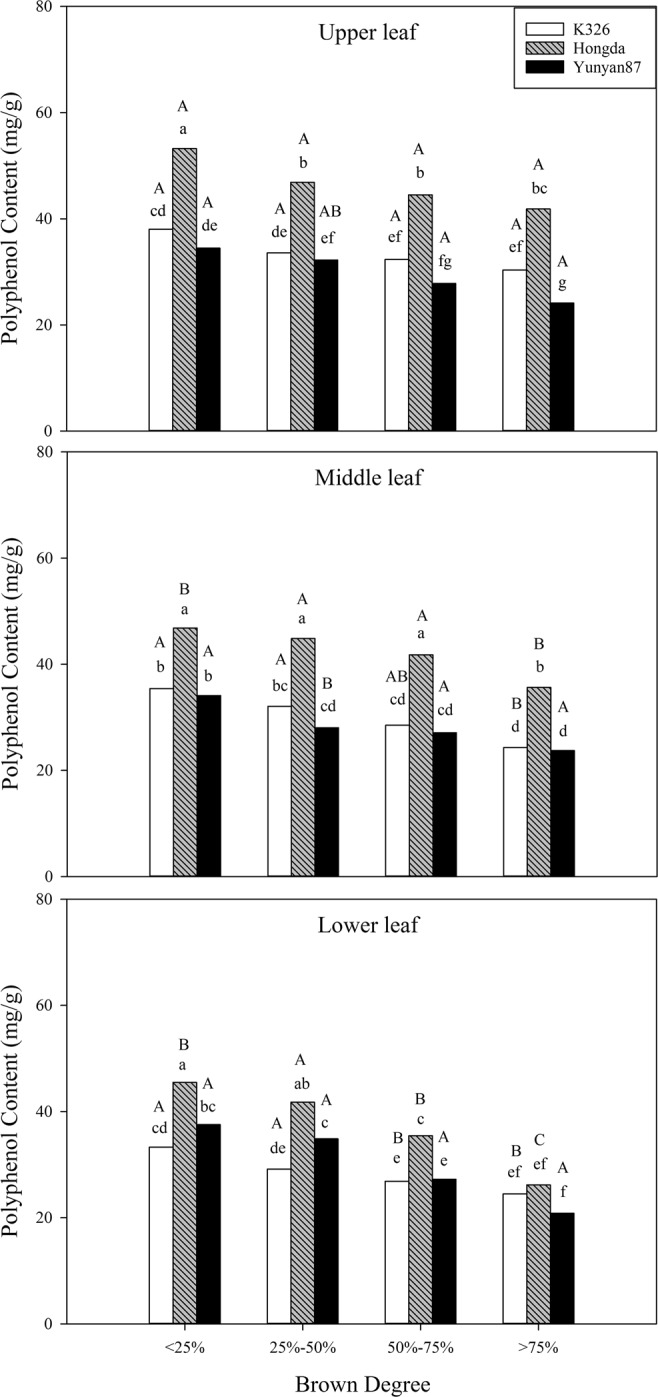
Effects of different BDs on polyphenol contents in flue-cured tobacco cultivars. Note: Different lowercase letters represent significant differences in polyphenol contents in tobacco leaves at same positions under combinations of different cultivars and BDs. Different capital letters denote the significant differences in polyphenol contents in tobacco leaves at different positions under combination of the same cultivar and BD.

As shown in Table 2, BD and position exert significant effects (*P* < 0.05) on the polyphenol content and BD has a synergistic effect with position. Figure 12 shows that the polyphenol content in different positions of the three flue-cured tobacco cultivars reduces with increasing BD.

When the BD is less than 25%, the polyphenol contents in upper leaves of the Hongda cultivar are significantly higher than those in middle and lower leaves. When BD ranges from 50% to 75%, the polyphenol contents in lower leaves of the K326 and Hongda cultivars are significantly lower than those in upper leaves. When the BD exceeds 75%, the polyphenol contents in middle and lower leaves of the K326 cultivar are significantly lower than that in upper leaves, and the polyphenol content in each position of the Hongda cultivar is significantly different.

### Effects of different BDs on industrial usability of different flue-cured tobacco cultivars

#### Effects of different BDs on shatter resistance index

**Figure 13:**
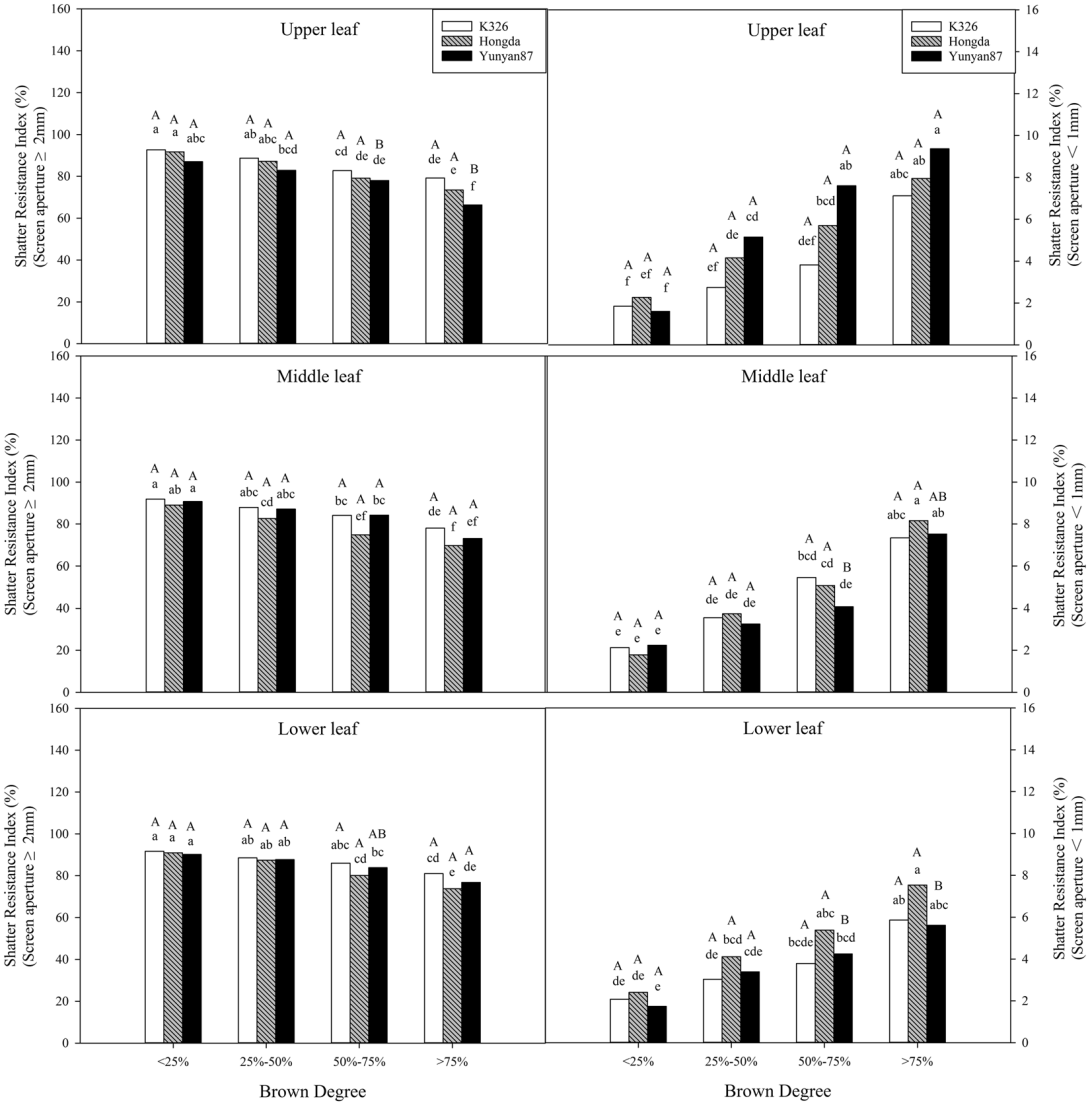
Effects of different BDs on shatter resistance index in flue-cured tobacco cultivars. Note: Different lowercase letters represent significant differences in shatter resistance index in tobacco leaves at same positions under combinations of different cultivars and BDs. Different capital letters indicate the significant differences in shatter resistance index in tobacco leaves at different positions under combination of the same cultivar and BD.

As shown in Table 3, BD, variety, and position exert significant effects (*P* < 0.05) on the shatter resistance index. BD has a synergistic effect with position. Figure 13 shows that the shatter resistance index (screen aperture < 1 mm) of tobacco leaves in different positions of each cultivar increase with increasing BD, while the shatter resistance index (screen aperture ≥ 2 mm) of tobacco leaves in different positions of each cultivar decrease with increasing BD. When the BD is less than 25%, the shatter resistance index (screen aperture ≥ 2 mm) in different positions of each cultivar is significantly greater than that when the BD exceeds 75%; however, when the BD is less than 25%, the shatter resistance index (screen aperture < 1 mm) in different positions of each cultivar is significantly lower than that when the BD exceeds 75%.

**Table 3:** Analysis of variance for the effects of browning degree, variety, position, and their interactions on industrial usability.

Effect/contrast	DF	Shatter resistance index (Screen aperture < 1 mm)	Shatter resistance index (Screen aperture ≥ 2 mm)	Sensory evaluation score
------------------------------Probability of a greater *F* value------------------------------				
Browning degree (B)	3	<0.0001	<0.0001	<0.0001
Variety (V)	2	<0.0001	<0.0001	0.0005
Position (P)	2	<0.0001	<0.0001	<0.0001
BV	6	0.1646	<0.0001	<0.0001
BP	6	0.0008	0.0401	0.7584
VP	4	<0.0001	<0.0001	<0.0001
BVP	12	0.0031	0.5087	<0.0001

#### Effects of different BDs on sensory evaluation score

**Figure 14:**
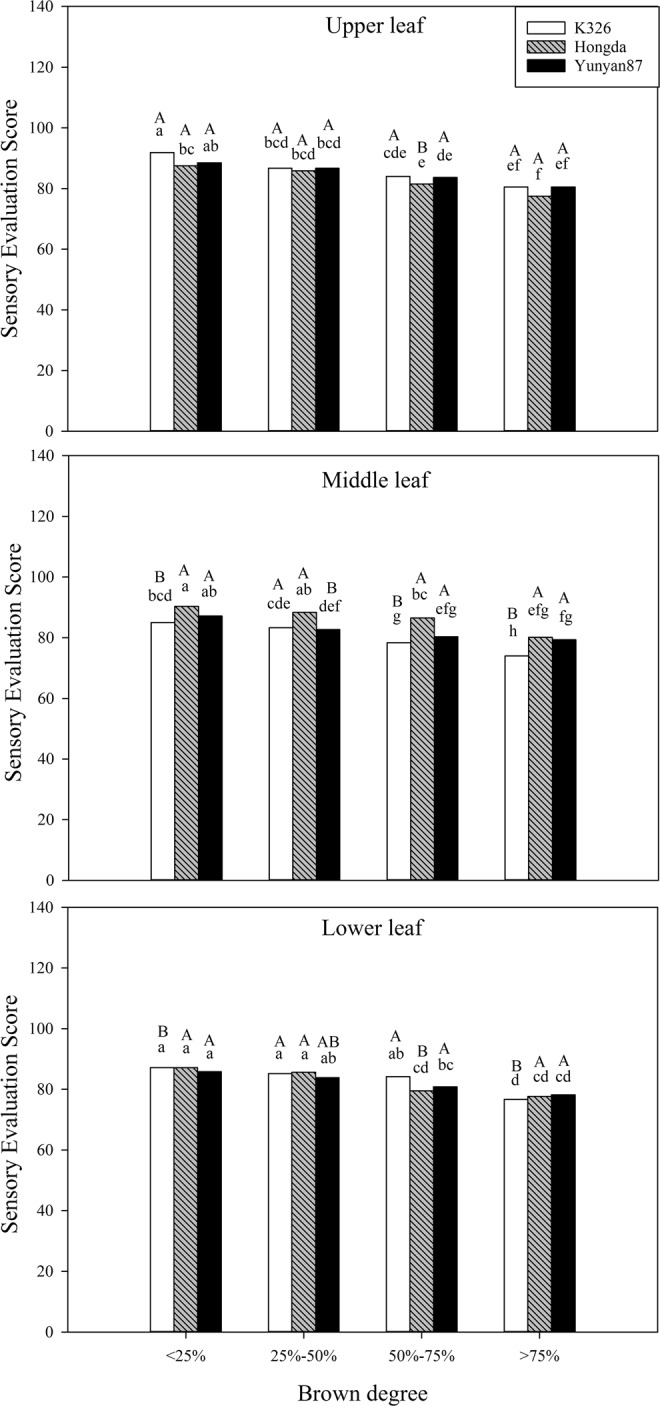
Effects of different BDs on sensory evaluation score of flue-cured tobacco cultivars. Note: Different lowercase letters represent significant differences in the sensory evaluation score of tobacco leaves at the same position under combinations of different cultivars and BDs. Different capital letters indicate the significant differences in the sensory evaluation score of tobacco leaves at different positions under combinations of the same cultivar and BD.

Based on Table 3, BD, cultivar, and position show significant influences on sensory evaluation score (*P* < 0.05). Furthermore, interactions of BD and cultivar, cultivar and position, and the three combined also significantly affect the sensory evaluation score. Figure 14 shows that the sensory evaluation score in each position of different cultivars decreases with increasing BD. At the same BD, when is less than 25%, the sensory evaluation score of upper leaves of the K326 cultivar is significantly higher than those of middle and lower leaves. When the BD ranges from 25% to 50%, middle and upper leaves of the Yunyan87 cultivar present significantly different sensory evaluation scores. For BDs between 50% and 70%, the sensory evaluation score of middle leaves of the K326 cultivar is significantly lower than those of upper and lower leaves, and the sensory evaluation score of middle leaves of the Hongda cultivar is significantly higher than those of upper and lower leaves. Furthermore, when the BD exceeds 75%, the sensory evaluation score of upper leaves of the K326 cultivar is significantly higher than those of middle and lower leaves.

### Extraction, isolation, and structure determination of the main chemical components of tobacco with grey speckles

**Figure 15:**
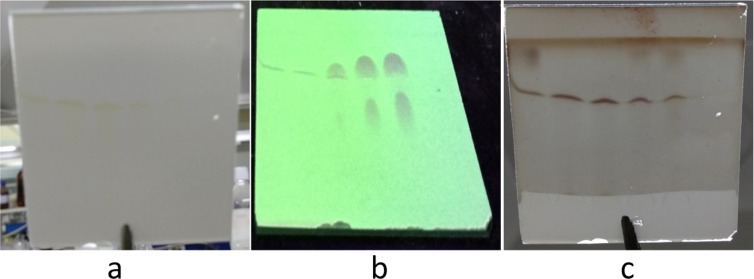
Thin-layer chromatogram of component Fr. Ab1a. Note: The sample was displayed under conditions such that the ratio of methylene chloride, methanol and formic acid was 5:1:0.1. (**a**) Thin-layer chromatogram without using H_2_SO_4_-EtOH; (**b**) Thin-layer chromatogram under ultraviolet light (254 nm) and (**c**) Thin-layer chromatogram after using H_2_SO_4_-EtOH.

**Figure 16:**
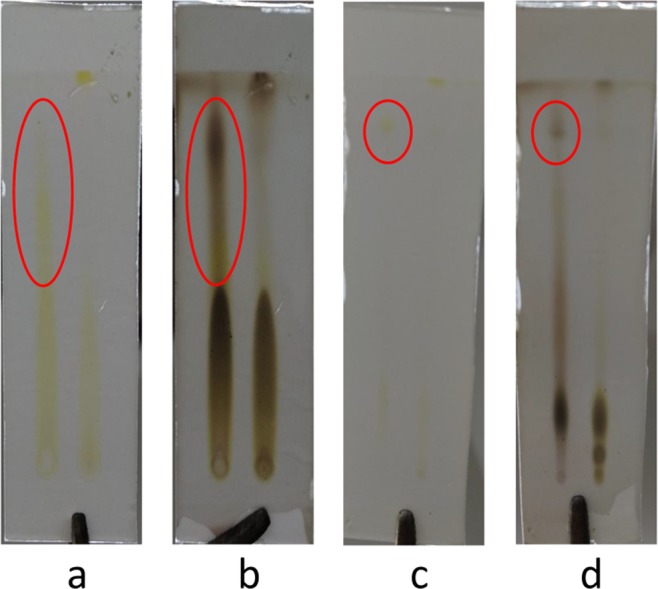
Thin-layer chromatograms of the tobacco samples with and without grey speckled leaves. Note: the left-hand sides of the figures show samples with grey speckles, while the right-hand sides show samples without grey speckles. (**a,c**) Thin-layer chromatograms without using H_2_SO_4_-EtOH, (**b,d**) Thin-layer chromatograms after using 5% H_2_SO_4_-EtOH. Conditions of (**a,b**) include a methylene chloride to methanol ratio of 5:1, while those in (**c,d**) include a methylene chloride: methanol: formic acid ratio of 5:1:0.1.).

Extracted solutions of the samples of leaves with and without grey speckles were analysed by using thin-layer chromatography. At a ratio of methylene chloride to methanol of 5:1 and a ratio of methylene chloride, methanol and formic acid of 5:1:0.1, ethanol extracts were separated and divided through a methylene chloride/methanol system and finally 220 mg of component Fr.Ab1a was separated and purified by utilising gel LH-20 (Fig. 15). Based on comparative analysis, it is known that parts with grey speckles and parts without grey speckles on tobacco leaves have different components (Fig. 16) and the components in the grey speckles are marked in red circles in Fig. 16. Sample Fr. Ab1a was separated using the Agilent1200 high-performance liquid chromatograph with an ultraviolet detector and YMC-pack ODS-A columns with a particle size of 5 μm and dimensions of 10 × 250 mm and passed through a system containing 35% MeCN/H_2_O solution at a flow rate of 2.0 mL/min. On this basis, 9.7 mg of the component 3-acetyl-6,7-dimethoxycoumarin (YC-ZJF) of grey speckles on leaves could be obtained (*t*_R_ = 20 min) (Fig. 2).

**Figure 17:**
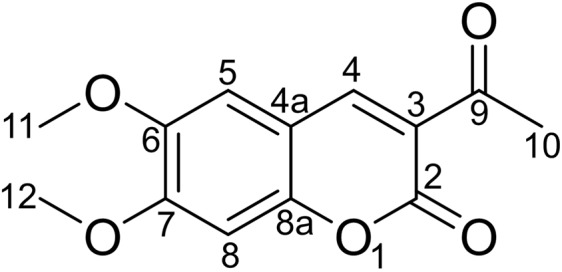
Chemical structure of component YC-ZJF from leaves with grey speckles.

**Figure 18:**
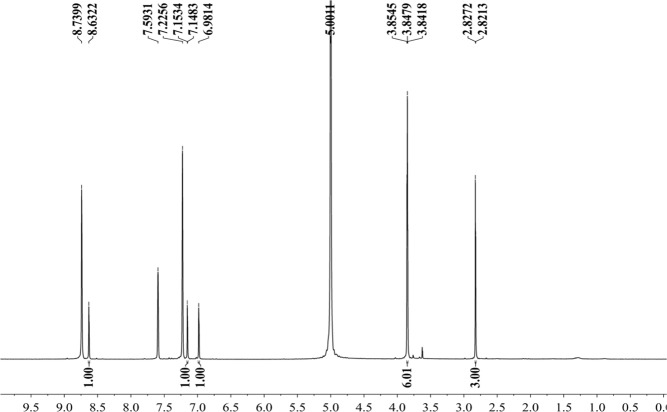
^1^H NMR spectrum of component YC-ZJF of leaves with grey speckles (400 MHz, pyrdine-*d*_5_).

**Figure 19:**
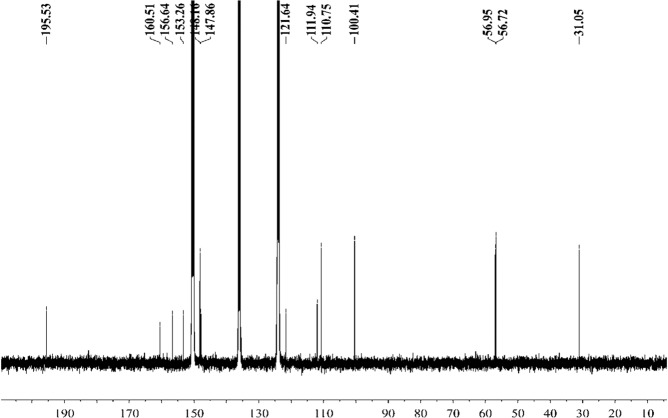
^13^C NMR spectrum of component YC-ZJF of leaves with grey speckles (100 MHz, pyrdine-*d*_5_).

**Figure 20:**
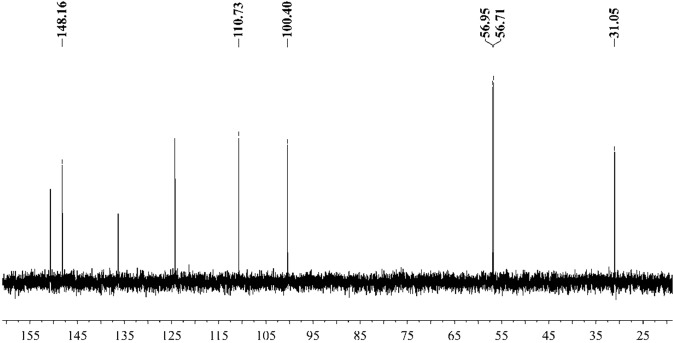
DEPT spectrum of component YC-ZJF of leaves with grey speckles (100 MHz, pyrdine-*d*_5_).

**Figure 21:**
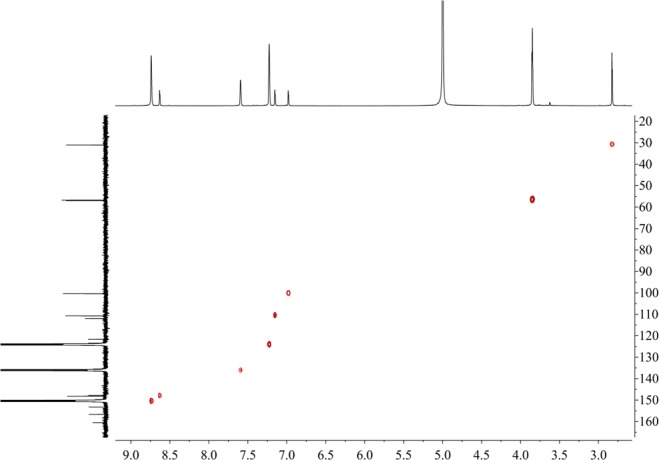
HSQC spectrum of component YC-ZJF of leaves with grey speckles (^1^H: 400 MHz, ^13^C:100 MHz, pyrdine-*d*_5_).

**Figure 22:**
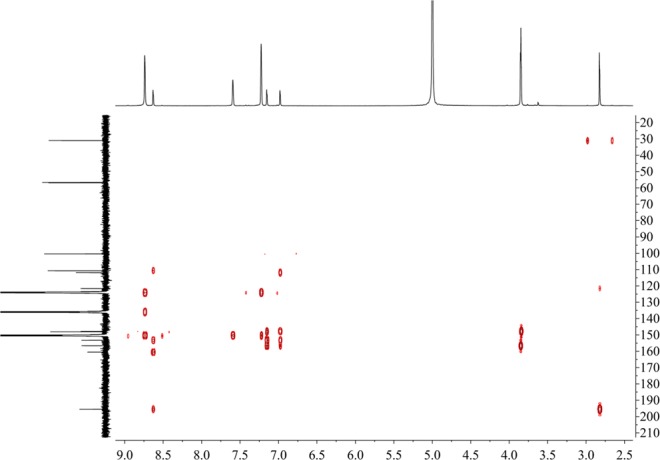
HMBC spectrum of component YC-ZJF of leaves with grey speckles (^1^H: 400 MHz, ^13^C:100 MHz, pyrdine-*d*_5_).

**Figure 23:**
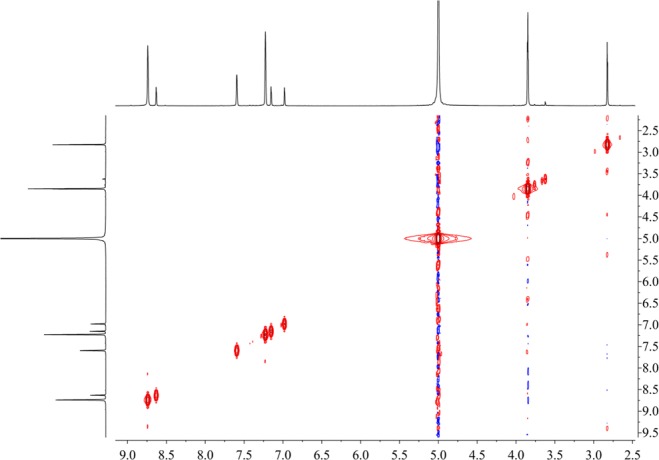
^1^H−^1^H COSY spectrum of component YC-ZJF of leaves with grey speckles (^1^H: 400 MHz, pyrdine-*d*_5_).

**Figure 24:**
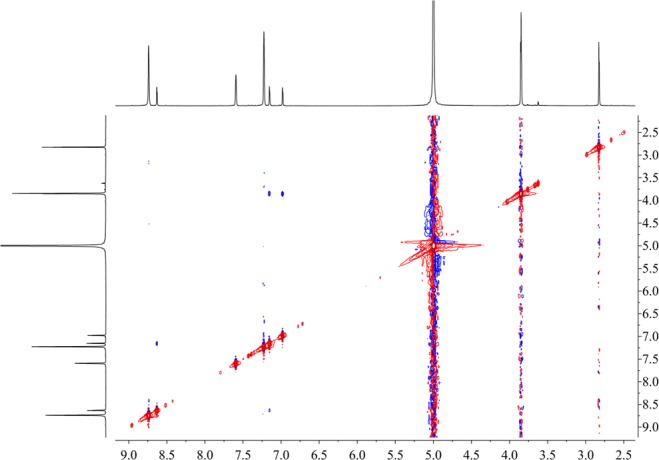
NOESY spectrum of component YC-ZJF of leaves with grey speckles (^1^H: 400 MHz, pyrdine-*d*_5_).

Figure 16 shows that the component from leaves with grey speckles has strong chromophores. After extracting extraction and separating tobacco with grey speckled leaves, the main chemical component, 9.7 mg of YC-ZJF was isolated from the tobacco with grey speckled leaves by repeated high-performance liquid chromatography (HPLC). The structure of the isolated compound (YC-ZJF) (Fig. 17) was determined to be 3-acetyl-6,7-dimethoxycoumarin as evinced by analysing its 1-d and 2-d NMR spectra (Figs. 18–24). Interestingly, 3-acetyl-6,7-dimethoxycoumarin was only reported as a synthetic compound, instead of a natural product (Takadate *et al*., 1995). Thus, 3-acetyl-6,7-dimethoxycoumarin should be an artifact during flue-curing of tobacco, therefore, 3-acetyl-6,7-dimethoxycoumarin should be one of the main components or related components in tobacco with grey speckled leaves.

3-Acetyl-6,7-dimethoxycoumarin (YC-ZJF) was isolated as a yellow amorphous powder. ^1^H NMR (400 MHz, C_5_D_5_N) *δ*_H_: 8.63 (s, H-4), 7.15 (s, H-5), 6.98 (s, H-8), 2.83 (s, CH_3_–10), 3.85 (s, 6-OCH_3_), 3.86 (s, 7-OCH_3_).^13^C NMR (100 MHz, C_5_D_5_N) *δ*_C_: 160.5 (C-2), 121.6 (C-3), 148.2 (C-4), 111.9 (C-4a), 110.8 (C-5), 147.9 (C-6), 156.6 (C-7), 100.4 (C-8), 153.3 (C-8a), 195.5 (C-9), 31.1 (C-10), 56.7 (C-11), 57.0 (C-12).

## Discussion

### Effects of different BDs on the economic value of different cultivars of flue-cured tobacco

Polyphenol oxidase and polyphenol substances are commonly found in plants and the browning reaction caused by polyphenol oxidase causes the loss of 50% of plant-based foods globally^27^. Besides tobacco, browning reactions in other plants generally cause direct damage, such as in cuts in apples, tomato, and mushroom preserved at room temperature^28–30^. There are many factors reflecting the quality of tobacco leaves, among which the proportion of superior tobacco directly reflects the economic value of tobacco leaves. In this experiment, the proportion of superior tobacco and output value of tobacco leaves in different positions of the three cultivars of flue-cured tobacco all decrease with increasing BD. The proportion of superior tobacco is an important index reflecting level of production technology used in the processing of tobacco leaves. According to the natural characteristics of tobacco plants, the theoretical maximum proportion of superior tobacco is 60% evaluated based on the number of leaves of a single plant^20^. Therefore, different BDs had different influences on the proportions of superior tobacco in different positions, however, these three cultivars all behaved such that the proportion of superior tobacco in the same position decreased with increasing BD. The output value is not only related to the quality of tobacco leaves, but is also correlated to the level of flue-curing technology used when processing tobacco leaves. Tobacco with grey speckled leaves has poor combustion characteristics, a low-quality aroma, and a small volume of aroma^31^. Moreover, it presents a strengthened biting taste and reduced quality. The higher the BD is, the lower the quality of tobacco leaves, so the output value also decreases.

### Effects of different BDs on chemical characteristics of different cultivars

#### Effects of different BDs on carbon metabolism conventional chemical components of different cultivars

Tobacco is an important economic crop. Its chemical composition is an important internal factor determining the quality of tobacco leaves and appropriate coordination of chemical components determines the value of tobacco^32^. The chemical components of tobacco leaves and their proportions decide the quality of tobacco leaves, thus exerting significant influence on the smoking quality of cigarettes^33^.

Decomposition, transformation, and accumulation of starch determine the quality and appearance rating of tobacco leaves^34^. Leffingwell^35^ believed that a high starch content in tobacco leaves during harvesting can cause imbalances in the chemical quality and the ratio of sugar to nicotine of flue-cured tobacco leaves and decrease their nicotine content. In addition, it can result in a smooth surface of cured tobacco leaves, raise proportions of tobacco leaves with miscellaneous colours and greenish tobacco leaves, and reduce industrial usability. In this experiment, with increasing BD, the starch contents in tobacco leaves in each position of the K326, Hongda, and Yunyan87 cultivars all significantly increase. On the whole, starch contents in upper and middle leaves of the three cultivars are significantly higher than those in lower leaves, however, for the Yunyan87 cultivar, when the BD ranges from 50% to 75%, the starch contents in lower leaves are significantly higher than those in middle and upper leaves. When the BD exceeds 75%, the starch contents in lower leaves of the Hongda cultivar are significantly higher than that in middle leaves. The BD affects the starch contents in leaves of different flue-cured tobacco cultivars, resulting in uncoordinated chemical compositions and low-quality flue-cured tobacco leaves.

Sugar content is an important index used when evaluating the quality of tobacco and its content reflects the carbon-supply capacity^36^. When sugar content of tobacco leaves is too low, the biting taste strengthens. If the sugar content is too high, the smoke is acidic, which influences the acid-base equilibrium inherent to smoking, so that smoke becomes tasteless and the tar content of smoke increases^37^. Based on analysis of the results, the reducing sugar contents in different positions of the three cultivars all decrease with increasing BD. Under the four BDs, the reducing sugar contents in lower leaves of the K326 and Hongda cultivars are relatively high, while a high reducing sugar content is found in middle and upper leaves of the Yunyan87 cultivar. For lower tobacco leaves of the K326 and Hongda cultivars, with the increase of BD, the reducing sugar content decreases more in comparison with that in upper leaves: however, with increasing BD, the reducing sugar contents in different positions of the Yunyan87 cultivar are reduced less compared with those in the K326 and Hongda cultivars, therefore, the BD exerts greater influences on the K326 and Hongda cultivars in comparison with that over the Yunyan87 cultivar and exerts greater influence on the reducing sugar content in lower leaves of the K326 and Hongda cultivars. The total sugar contents in different positions of the three cultivars all significantly decrease with increasing BD and, in particular, the decrease is greatest in middle leaves of the K326 cultivar. The reduction in total sugar content can affect the smoking quality of flue-cured tobacco, so that the biting taste increases and quality decreases^38^.

#### Effects of different BDs on nitrogen metabolism conventional chemical components of different cultivars

In the production of flue-cured tobacco, nitrogen is the most important nutrient element influencing yield and quality of tobacco leaves and the total nitrogen and alkaloid contents in flue-cured tobacco leaves reflect nitrogen-supply capacity^39^. In this experiment, the total nitrogen contents in tobacco leaves in each position of the Hongda and Yunyan87 cultivars do not show significant differences with the increase of BD, however, tobacco leaves in each position of the K326 cultivar show that the total nitrogen content increases significantly at high BD, therefore, the BD more significantly influences the total nitrogen content in the K326 cultivar than in the Hongda and Yunyan87 cultivars. Clarke^40^ found that the presence of alkaloids significantly affects the taste of tobacco and too high a nicotine content can increase the biting taste of tobacco. Under different BDs, the nicotine contents of tobacco leaves in different positions of the three cultivars are similar. The main factors affecting nicotine content are differences in cultivars and positions. For the K326 cultivar, nicotine contents in upper and middle leaves are significantly higher than that in lower leaves, while the Hongda and Yunyan87 cultivars demonstrate that nicotine contents in upper leaves are significantly higher than those in middle and lower leaves. On the whole, the three cultivars all contain the highest nicotine content in the upper leaves, with the lowest content in their lower leaves^41^.

#### Effects of different BDs on polyphenol components in different cultivars

Polyphenol substances have important influences on the physiological and biochemical activities of tobacco, the colour and lustre of tobacco leaves, the aroma and taste of cigarettes, and their physiological strength^42^. In particular, as an important aroma precursor of flue-cured tobacco, it can be decomposed into a variety of aroma substances and be combined with proteins or be catalysed by polyphenol oxidase under the browning reaction^43^. This study showed that, with increased BD, the polyphenol content decreases. Chlorogenic acid and rutin are the main components of polyphenol substances. Sun *et al*.^44^ demonstrated that the chlorogenic acid content is mainly affected by cultivar, followed by interaction between altitude and cultivar. Our result also showed that cultivar exerts significant effects (*P* < 0.05) on the polyphenol content and cultivar has a synergistic effect with BD and position.

### Effects of different BDs on other industrial indices of different cultivars

#### Effects of different BDs on shatter resistance index of different cultivars

The physical resistance to further processing of tobacco leaves is the focus of enterprises involved in re-drying tobacco and the better the physical resistance to further processing, the less tobacco is lost during defoliation^45^. The data show that the physical resistance to further processing, as represented by shatter resistance, decreases with increasing yellowing. The shatter resistance index (screen aperture <1 mm) of tobacco leaves in different positions of each cultivar increase with increasing BD, while the shatter resistance index (screen aperture ≥2 mm) of tobacco leaves in different positions of each cultivar decrease with increasing BD, which indicates that the capability of tobacco leaves to resist shattering decreases with increasing BD.

#### Effects of different BDs on sensory qualities of different cultivars

Tobacco is meant to be flavoursome and its quality is mainly evaluated through smoking; in this experiment, the sensory qualities of the three cultivars all decrease with increasing BD. Under each BD, the sensory quality of upper leaves of the K326 cultivar is higher than those in other positions. Moreover, the sensory qualities of middle leaves of the Hongda cultivar under each BD are higher than those of the other two cultivars. The sensory evaluation score is affected by chemical components, such as starch, total sugar, total nitrogen, and nicotine^46^. The influences of chemical components of flue-cured tobacco on sensory quality are generally explained through the theory of sugar-alkali equilibrium^47^. When tobacco leaves contain the ideal chemical components, the sensory quality of tobacco leaves is relatively high. For example, it is appropriate that the total sugar content is 20% to 28% and the difference between the contents of two sugars is less than 5%^48^. The experimental results show that the reducing sugar and total sugar contents in tobacco leaves in different positions of the three cultivars all decrease with increasing BD and the total sugar content is significantly positively correlated with sensory quality.

### Discussion of structure identification of substances of grey speckles on leaves of flue-cured tobacco

Previous research into the causes of formation of tobacco with grey speckled leaves mainly focus on polyphenol oxidase and polyphenol substances^49^. Our previous study clarified that the identity of the bridging oxygen must be a water molecule in the tyrosinase which is a typical polyphenol oxidase^50^ purified from a new polyphenol oxidase called PPO II found in tobacco and proved to accumulate in the injured parts of tobacco leaves. The poor quality of fresh tobacco leaves is one of causes of the appearance of tobacco with grey speckled leaves. If fresh tobacco leaves are infected by disease, much polyphenol oxidase accumulates therein and tobacco with grey speckled leaves become more likely during the curing process^51^. This experiment found that the structure of the component derived from the tobacco leaves with grey speckles is YC-ZJF, which was previously reported as a synthetic compound, instead of a natural product; however, due to the complexity of the formation of grey speckles, it is difficult to conclude that YC-ZJF is the major component of grey speckles. It is also possible that YC-ZJF might be an intermediate if grey speckles are largely composed of macromolecules. Nonetheless, the identification of the structure of YC-ZJF is still important to the further elucidation of the mechanisms of formation of tobacco with grey speckled leaves. Meanwhile, according to the identified structure of the substance, the corresponding components capable of reducing the presence of the substance forming grey speckles on tobacco leaves can be investigated, so as to reduce the incidence of tobacco with grey speckled leaves in the flue-curing process.

## Conclusion

The effects of the browning reaction of flue-cured tobacco on tobacco value and industrial usability were elucidated along with the structural identification of grey matter thereon. With increasing BD, the proportion of superior tobacco and output value of tobacco leaves decrease regardless of cultivar or position. For carbon metabolism, the starch is not degraded with increased BD, and total sugar and reducing sugar contents decrease accordingly. For nitrogen metabolism, the protein, total nitrogen, and nicotine contents change albeit slightly. The industrial indices, including shatter resistance index and smoking quality decrease with increasing BD. More importantly, the chemical structure of the main components of tobacco with grey speckled leaves was identified and the main component of the separated parts with grey speckles was determined to be 3-acetyl-6,7-dimethoxycoumarin. These results demonstrated that the increase of BD reduces the economic value and industrial usability of tobacco. In the planting and production of tobacco, it is particularly important to reduce the incidence of tobacco with grey speckled leaves.

## Data Availability

All data generated or analysed during this study are included in this published article (and its Supplementary Information files).
